# Perspectives on childhood coronavirus disease vaccination in Japan and influencing factors

**DOI:** 10.1111/ped.15819

**Published:** 2024-09-30

**Authors:** Madoka Lelliott, Masatsugu Sakata, Ayako Kohno, Rie Toyomoto, Ayuko Matsumoto, Toshi A. Furukawa

**Affiliations:** ^1^ Department of Health Promotion and Human Behavior Kyoto University Graduate School of Medicine and School of Public Health Kyoto Japan; ^2^ Internationalization Promotion Office, School of Public Health, Graduate School of Medicine Kyoto University Kyoto Japan; ^3^ Department of Education Koyasan University Wakayama Japan

**Keywords:** child health, coronavirus, immunization, parental, vaccine hesitancy

## Abstract

**Background:**

To support parental decision‐making it is important to understand parents' perspectives on vaccination for their children and the factors that contribute to their vaccine hesitancy. There have been relatively few studies in this area in Japan, particularly with longitudinal and mixed methodologies.

**Methods:**

We used an explanatory sequential mixed methods approach to describe longitudinal changes in vaccine acceptance and to explore factors associated with parental coronavirus 2019 (COVID‐19) vaccine hesitancy. We recruited parents who had children aged 6 months to 11 years old from five facilities in Japan. Two cross‐sectional online surveys and semi‐structured online interviews were conducted. Logistic regression analysis was used to explore factors associated with parents' vaccine hesitancy for their children, and thematic analysis was used to analyze the interview data.

**Results:**

In total, 134 parents responded to both online surveys and, of those, 10 participated in interviews. Acceptance rates of COVID‐19 vaccination for their children were 19.4% (26/134) at the first survey and 11.2% (15/134) at the second survey. Integration of the data identified that the main factors for vaccine hesitancy included vaccine safety, vaccine effectiveness, government policy, and recommendations from people close to parents.

**Conclusions:**

Readily available and more balanced information, and community‐wide support from people close to parents and familiar health‐care providers are likely to provide better support for parents' decision‐making. Further investigation is required on how to provide information in an easily understood manner.

## INTRODUCTION

The development of vaccines for coronavirus disease (COVID‐19) has been critical in minimizing the impact of the pandemic. In Japan, COVID‐19 vaccination started in February, 2021 for healthcare workers and May/June, 2021 for general adults, and was expanded to those over 5 years old from February, 2022 and over 6 months old to 5 years old from October, 2022. At the time of this study, children aged 6 months to under 5 years in Japan were recommended to receive COVID‐19 vaccination in a two‐dose schedule for the Moderna monovalent Omicron XBB.1.5 vaccine and in a three‐dose schedule for the Pfizer monovalent Omicron XBB.1.5 vaccine. Children over 5 years old were recommended to receive the Moderna or Pfizer COVID‐19 vaccine in a two‐dose schedule. Vaccination was provided free for all Japanese citizens. Vaccinations were available at medical institutions and vaccination sites in local municipalities. (COVID‐19) vaccination for children is important for their health, emotional well‐being and facilitates participation in social activities.^1^ Previous studies showed 42.9% of parents who had children aged between 0 and 15 years^2^ and 64.7% of parents who had children aged 3–14 years intended to vaccinate their children.^3^ However, in January 2024, only 4.4% of children under 5 years old and 24.5% of children over 5 years old received one or more COVID‐19 vaccinations.^4^

In association with widespread vaccination, vaccine hesitancy has surfaced as a key issue.^5^ The World Health Organization identified it as one of the top 10 threats to global health and included it in the 5‐year strategic plan in 2019.^6^ Vaccine hesitancy is defined as a “delay in acceptance or refusal of vaccination despite availability of vaccination services.”^7^ The main factors influencing vaccine hesitancy consist of three categories (confidence, complacency, and convenience) in the 3Cs model.^7^ Confidence refers to trust in the effectiveness and safety of vaccines, the system that delivers them, and the motivations of policy‐makers. Complacency refers to situations when people assess the risks of infection to be low and the vaccine unnecessary. Convenience includes factors such as geographical accessibility and health literacy. These factors are complex and situation dependent. Previous studies have shown that the Japanese population is generally highly skeptical regarding the safety and effectiveness of vaccines.^8^, ^9^ This was especially true for childhood vaccination, with strong parental hesitation to vaccinate their children.^10^, ^11^, ^12^ Unlike models adopted in other countries, there are no school vaccination programs in Japan (including for COVID‐19). The added burden of visiting a medical institution or vaccine site suggests that lack of convenience may contribute toward hesitancy in Japan. Moreover, as was the case with the HPV vaccine,^13^ negative information about COVID‐19 vaccination on social media platforms might affect complacency.

Studies of parents' perspectives on COVID‐19 vaccination for their children have been conducted in several countries.^14^, ^15^, ^16^, ^17^ However, the majority of studies to date have been conducted as quantitative, mostly cross‐sectional, studies. Longitudinal studies are necessary as perspectives on vaccination are prone to change in a short timeframe.^18^ Furthermore, as a comprehensive approach to understand the vaccine hesitancy issue, mixed methods research can reveal additional details for each variable used in quantitative methods.^19^, ^20^ However, there is little evidence derived from these approaches examining Japanese parental COVID‐19 vaccine hesitancy factors.^2^, ^3^, ^21^

In this descriptive study we therefore aimed to investigate longitudinal changes in acceptance and hesitancy about COVID‐19 vaccination and examine factors influencing COVID‐19 vaccine hesitancy among parents, using an explanatory sequential mixed methods approach. This research contributes to the understanding of how to support parental decision‐making, and highlights avenues for further research into these issues.

## METHODS

**FIGURE 1:**
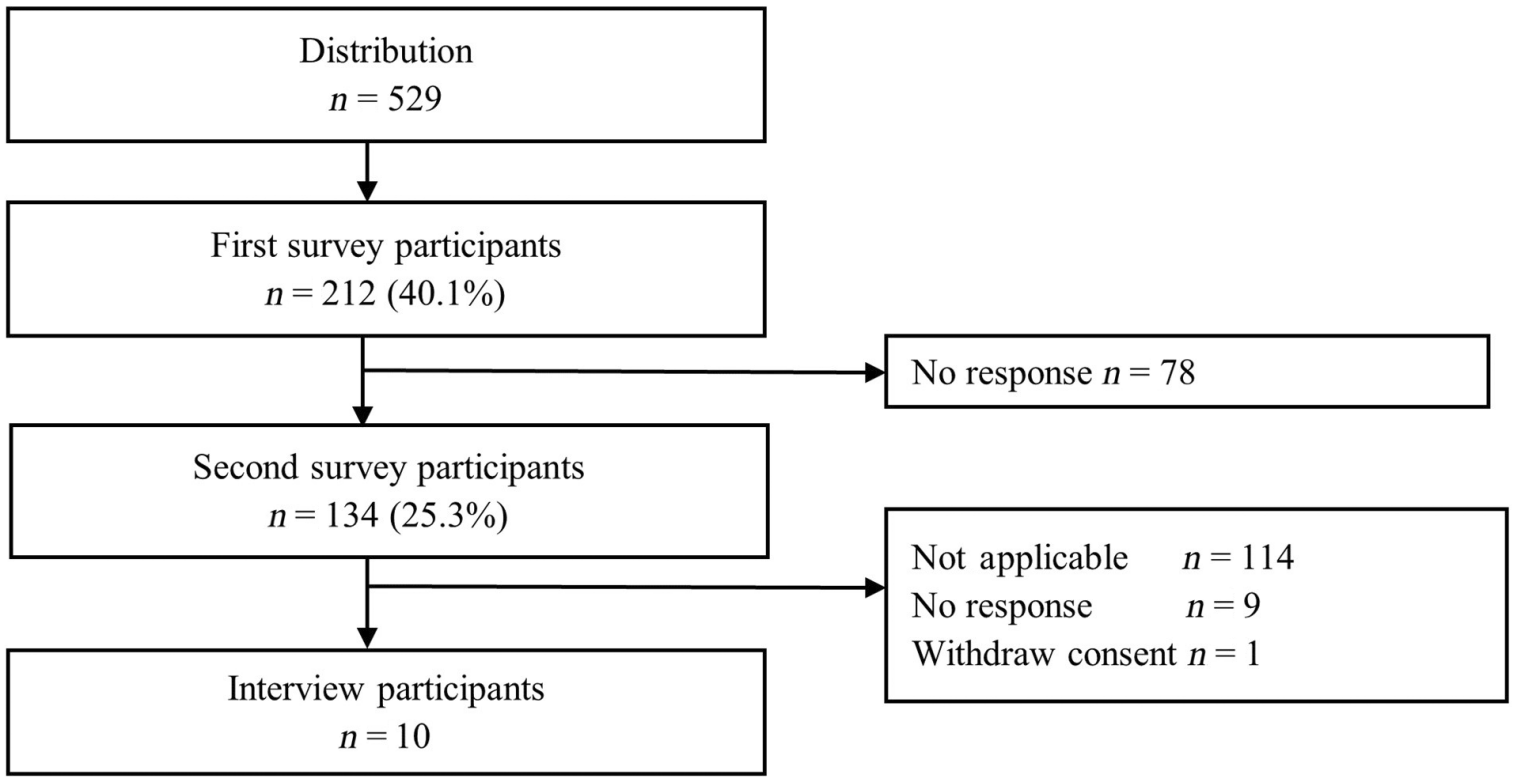
Process of collecting quantitative and qualitative data

We used an explanatory sequential mixed methods approach,^20^ which included quantitative methods (two cross‐sectional online surveys) and qualitative methods (interviews). Figure 1 shows the process of collecting quantitative and qualitative data.

Our paradigm stance for mixed methods was pragmatism, which combined deductive and inductive ways to mix qualitative and quantitative data in the study process.^20^ We adhered to the following guidelines to report this study: Strengthening the Reporting of Observational Studies in Epidemiology^22^, Consolidated Criteria for Reporting Qualitative Research^23^ and “A checklist of mixed methods elements in a submission for advancing the methodology of mixed methods research” in the *Journal of Mixed Methods Research*.^24^

### Quantitative study

#### Sampling

We recruited participants from five facilities (two public day‐care centers, and one private and two public after‐school centers) located in Osaka and Kyoto prefectures in Japan. To take part in the survey, parents had to have a child aged 6 months to 11 years old at the time of informed consent (IC), be 18 years old or older, and understand Japanese. Parents had to register their email address for follow up. The sample size (*n* = 185) was calculated using the formula

$$n=\frac{Z^{2}P\left(1-P\right)}{d^{2}}$$

with *Z* = 1.96 for a 95% confidence interval (CI), *d* = 5% precision^25^ and *P* = 14 % estimated vaccination refusal rate.^2^

We conducted the first survey between January 24 and February 13, 2022 and the second survey between April 18 and May 14, 2022. This was before and after the vaccination program started for children over 5 years old and before the vaccination program for children under 5. To recruit for the first survey, we distributed advertisements stating the purpose and details at each facility. Survey leaflets were distributed by facility staff at two facilities, and electronic notifications via application software at three facilities were sent to all parents who had a child affiliated with the facility at the start of the survey. For the second survey, eligible parents also received several reminder emails from the authors. Surveys were anonymous and administered via Google Forms. Informed consent was obtained in the questionnaire and agreed to before starting the response. No incentives were provided to participants.

#### Questionnaire

The questionnaire was developed based on the 3Cs model^7^ and recent studies on vaccine hesitancy^19^, ^26^ (Supporting Information, Appendix S1). Parents answered questions about vaccine hesitancy factors and were given four options as an answer, “no,” “probably no,” “probably yes,” and “yes.” After vaccination for children over 5 years old started, we added two questions in this section: (i) whether parents thought the time for vaccination was appropriate or not and (ii) whether parents thought the place for vaccination was appropriate. To assess parents' perspective on COVID‐19 vaccination for their children, they answered the question “Do you want your child to get COVID‐19 vaccination if it is available?” They were given three options: “no,” “undecided,” and “yes.” For the second survey, the answer “vaccinated” was added as an option. In the first survey, they also answered three other questions to assess their perspective of other vaccines— human papillomavirus (HPV), mumps and influenza. Finally, they answered questions regarding their sociodemographic characteristics such as age, gender, education, employment, number of children, health‐care professionals (HCPs) and educators in the family and COVID‐19 and influenza vaccine history, and their children's characteristics such as age, gender, birth order, chronic disease status, vaccination status in immunization programs and influenza vaccine history. We conducted pilot studies with several parents who met the inclusion criteria using the same survey format as for the main study. Feedback was obtained by post‐survey interviews. We modified survey questions based on feedback from pilot studies to improve comprehension of questions.

#### Data analysis

Based on the definition of vaccine hesitancy, parents who answered “no” or “undecided” to the question “Do you want your child to get COVID vaccination if it is available?” were defined as the “hesitancy group,” whereas parents who answered “yes” were defined as the “acceptance group.” In this study, the change to acceptance rate was defined as the proportion of parents who hesitated about vaccination at the first survey and moved to acceptance at the second survey. The change to hesitancy rate was defined as the proportion of parents who accepted vaccination at the first survey and moved to hesitancy at the second survey.

We included vaccine safety, vaccine efficacy, safely manage and supply, agree with government policy, recommendations from people close to parents, trust in information from television/social networking services/people close to parents/HCPs as confidence factors in the 3Cs model. As complacency factors, we asked how parents felt prevention measures, the likelihood of acquiring COVID‐19 in group/non‐group life, worry about the symptom. As convenience factors, we also asked appropriateness of time and place. For other questions about vaccines, if parents answered “no” or “probably no,” this was grouped into a single category “no.” In the same way, if parents answered “yes” or “probably yes,” this was grouped into the category “yes”.

This was an exploratory study. Logistic regression analysis was used to identify factors associated with parents' vaccine hesitancy for their children. We calculated odds ratios (ORs) in 33 variables with 95% CIs. As multiple tests increase Type I errors, we did not report *p*‐values, and additionally set a cut‐off for the interpretation of effect sizes, OR >3.0 or <0.33.^27^ To calculate ORs, we added a fixed value of 0.5 to all cells of study results tables if any cell had a value of zero.^28^ Analyses were conducted using R version 4.1.2 and Microsoft Excel version 2210.

### Qualitative study

#### Sampling

We selected interviewees from parents who completed both the first and second surveys and who were hesitant for COVID‐19 vaccination for their children at the first survey and/or second survey. We adopted intensity sampling, which is a type of purposeful sampling approach.^29^ Recruitment was conducted, and informed consent was obtained, by email.

#### Data collection

Semi‐structured online interviews were conducted from May to June, 2022, using an interview guide that was focused on the research question: “What are parental perceptions regarding their decisions on COVID‐19 vaccination for their children?” We conducted two pilot interviews with parents who met the inclusion criteria and modified questions based on their feedback to improve ease of understanding (Supporting Information, Appendix S2). We did not include data from pilot interviews in the analysis.

The lead author (ML—a nurse) conducted interviews through phone, Zoom or LINE (a freeware communication app), according to the preferences of parents. In line with the mixed metods strategy of reflexivity, the interviewer informed parents in advance that she was a nurse. She encouraged participants to discuss their opinions freely. Data from interviews were audio recorded and transcribed verbatim, maintaining the anonymity of the participants. Field notes were also created after each interview.

#### Data analysis

Transcripts were imported and managed in MAXQDA 22.2.0. The data were analyzed using thematic analysis.^30^ Investigator triangulation was used to achieve rigor. Three authors (ML, MS—a psychologist, and RT—a midwife) read all transcripts independently and discussed coding. The lead author categorized themes and subthemes and then discussed them with co‐authors until discrepancies were resolved. After analysis, transcripts were rechecked to ensure categories accurately represented what the participants had said.

### Integration

Mixed‐methods integration was performed using data from the quantitative study to inform the qualitative study, and then by using these connected results to draw conclusions.^20^ In the first stage of integration, we recruited interview participants based on survey results. The interview guide was also modified based on survey results to better focus on parents who indicated hesitancy. In the second stage, the two sets of data were integrated by considering how the qualitative findings explained and extended specific quantitative findings with a joint display (Table 1). By using meta‐inferences in the joint display, we derived overall conclusions from quantitative and qualitative results. The co‐authors checked these procedures to ensure their validity and coherence.

**TABLE 1:** Integration of main findings across quantitative and qualitative studies

Quantitative findings	Qualitative findings	Meta‐inferences
When parents worry about safety of the vaccine, they do not intend to vaccinate their children. **First survey** OR: 22.1 (6.12–142.3) **Second survey** OR: 12.4 (3.22–81.5)	**Concern about long‐term unknown side effects** “In particular, my children are still going to live for a long time to come, so I'm worried about what kind of side effects they will get. Even from a long‐term perspective, it's a really unknown vaccine, so I'm worried.” (ID9) **Concern about side effects experienced by parents** “I got vaccination twice, well, I got fatigue and fever after the second time. I'm worried about whether even my child can endure this feeling.” (ID52) **Uncertainty of information about childhood vaccination** “From the beginning, it is not like ‘there are such side effects, but there are such merits’. Only merits are mentioned.” (ID9)	Concern about future illness and infertility due to side effects was a reason parents hesitate for children's COVID‐19 vaccinations. Reasons for worrying about long‐term effects included that the vaccine called mRNA is new type of vaccine, the short period since the start of vaccination, and past reports of adverse side effects of HPV vaccines. If parents and/or someone close to parents suffered adverse side effects, parents worried that their children would have the same experience. If their children have allergies, parents tended to hesitate for vaccination. Unbalanced information and lack of information about safety led to hesitation. Uncertainty in safety caused parental vaccine hesitancy.
When parents suspected the vaccine was ineffective, they do not intend to vaccinate their children. **First survey** OR: 14.1 (2.82–257.2) **Second survey** OR: 10.5 (2.01–193.4)	**Unreliable effectiveness** “I just feel a sense of discomfort. Third time, fourth time (of vaccination) … I wonder how they will go…I wonder if it really works…” (ID11)	Some parents recognized that the vaccine effectiveness was short‐term and were skeptical about vaccine effectiveness itself. Taking the same vaccine several times caused doubts about vaccine effectiveness.
When parents do not agree with government policy, they do not intend to vaccinate their children. **First survey** OR: 6.13 (1.98–26.9) **Second survey** OR: 18.3 (1.07–312.9)	**Ambiguous explanation from government authorities** “HPV vaccination was promoted, and (children) got, but there were a lot of side effects and then it was canceled. […] But it's been promoted again recently, isn't it? […] I think the reason why it was promoted again is a little vague. That's why I still feel distrust toward the government.” (ID43) **Voluntary and insufficient compensation** “Government says like vaccination is voluntary. It seems like ‘you got vaccination with your own will’. I understand there is not so much compensation for vaccination.” (ID11)	Some parents felt government explanations about not only vaccine policy but also the entire COVID‐19 infection policy and past vaccine measures, such as HPV vaccination, lacked transparency. Voluntary nature and lack of compensation for vaccination was mentioned as an issue.
When parents do not have recommendations from people close to parents, they do not intend to vaccinate their children. **First survey** OR: 14.3 (4.99–52.0) **Second survey** OR: 28.7 (7.26–192.0)	**No encouragement from close personal contacts** “Same with my husband and mother, we all knew some children got side effects after starting children's vaccination…That's why they worry. They were people who took the stance that if they could get vaccination, it would be better to do it.” (ID43)	Parents were reluctant to vaccinate their children due to lack of encouragement from family members, such as spouses and grandparents, and from other close people.

Abbreviations: COVID‐19, coronavirus disease; HPV, human papillomavirus; mRNA, messenger RNA.

Data collection, analysis, and integration were conducted in Japanese. English translations were perfomed and agreed upon by the study authors.

## RESULTS

### Quantitative findings

#### Sample characteristics

We distributed invitations to complete the survey to 529 households; 40.1% (212) of parents answered the first online questionnaire survey and 25.3% (134) of parents completed both the first and second online questionnaire survey (Figure 1). Table 2 shows their baseline sociodemographic characteristics. There were no differences in sociodemographic characteristics and vaccine acceptance and hesitancy rate in the first survey results between parents who completed the second survey and those that dropped out.

**TABLE 2:** Sociodemographic characteristics and factors associated with hesitation related to COVID‐19 vaccination of children in the first survey in Japan, 2022 (*n* = 134)

	No. (%)	Acceptance group, *n* = 26 (%)	Hesitancy group, *n* = 108 (%)	OR^a^ (95% CI)
Parents characteristics				
Gender				
Father	11 (8.2)	3 (11.5)	8 (7.4)	Ref
Mother	123 (91.8)	23 (88.5)	100 (92.6)	1.63 (0.34–6.14)
Age (years)				
25–39	75 (56.0)	10 (38.5)	65 (60.2)	Ref
≥40	59 (44.0)	16 (61.5)	43 (39.8)	0.41 (0.17–0.98)
Educational level				
High school or below	29 (21.6)	10 (38.5)	19 (17.6)	Ref
Higher than high school	105 (78.4)	16 (61.5)	89 (82.4)	2.92 (1.13–7.42)
Employment status				
Employed	120 (89.6)	23 (88.5)	97 (89.8)	Ref
Unemployed	14 (10.4)	3 (11.5)	11 (10.2)	0.87 (0.25–4.07)
Number of children				
1	45 (33.6)	10 (38.5)	35 (32.4)	Ref
2	60 (44.8)	12 (46.2)	48 (44.4)	1.14 (0.44–2.95)
≧3	92 (21.6)	4 (15.4)	25 (23.1)	1.79 (0.53–7.10)
At least one HCP in family				
Yes	24 (17.9)	7 (26.9)	17 (15.7)	Ref
No	110 (82.1)	19 (73.1)	91 (84.3)	1.97 (0.68–5.23)
At least one educator in family				
Yes	17 (12.7)	5 (19.2)	12 (11.1)	Ref
No	117 (87.3)	21 (80.8)	96 (88.9)	1.90 (0.56–5.76)
Received influenza vaccine this season				
Yes	70 (52.2)	15 (57.7)	55 (50.9)	Ref
No	64 (47.8)	11 (42.3)	53 (49.1)	1.31 (0.56–3.18)
Received COVID‐19 vaccine				
Yes	120 (89.6)	26 (100)	94 (87.0)	Ref
No	14 (10.4)	0 (0)	14 (13.0)	8.13 (0.47–140.9)
Influenza vaccine is important				
Yes/probably yes	99 (73.9)	22 (84.6)	77 (71.3)	Ref
No/probably no	35 (26.1)	4 (15.4)	31 (28.7)	2.21 (0.77–8.03)
Mumps vaccine is important				
Yes/probably yes	117 (87.3)	24 (92.3)	93 (86.1)	Ref
No/probably no	17 (12.7)	2 (7.7)	15 (13.9)	1.94 (0.50–12.8)
HPV vaccine is important				
Yes/probably yes	81 (60.4)	20 (76.9)	61 (56.5)	Ref
No/probably no	53 (39.6)	6 (23.1)	47 (43.5)	2.57 (1.00–7.48)
Children's characteristics				
Gender				
Boy	61 (45.5)	12 (46.2)	49 (45.4)	Ref
Girl	73 (54.5)	14 (53.8)	59 (54.6)	1.03 (0.43–2.44)
Age (years)				
1–4	49 (36.6)	12 (46.2)	37 (34.3)	Ref
5–11	85 (63.4)	14 (53.8)	71 (65.7)	1.64 (0.68–3.93)
Birth order				
1	95 (70.9)	20 (76.9)	75 (69.4)	Ref
2	30 (22.4)	2 (7.7)	28 (25.9)	3.73 (1.00–24.3)
≧3	9 (6.7)	4 (15.4)	5 (4.6)	0.33 (0.08–1.45)
Child has a chronic disease				
Yes	13 (9.7)	2 (7.7)	11 (10.2)	Ref
No	121 (90.3)	24 (92.3)	97 (89.8)	0.73 (0.11–2.98)
Received all available vaccines				
Yes	123 (91.8)	26 (100)	97 (89.8)	Ref
No	11 (8.2)	0 (0)	11 (10.2)	6.25 (0.36–109.6)
Received influenza vaccine this season				
Yes	73 (54.5)	15 (57.7)	58 (53.7)	Ref
No	61 (45.5)	11 (42.3)	50 (46.3)	1.18 (0.50–2.85)

Abbreviations: %, percentage; CI, confidence interval; HCP, Health care professional; HPV, Human papillomavirus; *n*, number; OR, odds ratio.

^a^ Higher odds ratio values indicate more hesitation.

#### Vaccine acceptance and hesitancy

In the first survey, 19.4% (26/134) (95% CI: 12.7–26.1) of parents indicated they would vaccinate their children for COVID‐19, 32.8% (44/134) (95% CI: 24.9–40.8) indicated they would not, and 47.8% (64/134) (95% CI: 39.3–56.2) were undecided. In the second survey, only 11.2% (15/134) (95% CI: 5.9–16.5) of parents indicated they would vaccinate—this included only one parent whose child had already been vaccinated; 47.0% (63/134) (95% CI: 38.6–55.5) of parents were not planning to vaccinate, and 41.8% (56/134) (95% CI: 33.4–50.1) of parents were undecided. Of parents who were hesitant about their child's vaccination in the first survey, 4.6% (5/108) (95% CI: 0.7–8.6) changed to accept their child's vaccination in the second survey, whereas 61.5% (16/26) (95% CI: 42.8–80.2) of parents who accepted vaccination in the first survey changed to become hesitant in the second survey.

#### Factors associated with COVID‐19 vaccine hesitancy

Table 2 and 3 show the results of binary logistic regression analysis. Parents' and their children's sociodemographic characteristics did not differ significantly between the acceptance or hesitancy group (0.33 < OR < 3.0). Vaccine safety (the first survey OR: 22.1 [6.12–142.3], the second survey OR: 12.4 [3.22–81.5]), vaccine effectiveness (the first survey OR: 14.1 [2.82–257.2], the second survey OR: 10.5 [2.01–193.4]), safe management and supply (the first survey OR 5.51 [1.52–35.5], the second survey OR 6.83 [1.30–125.9]), government policy (the first survey OR: 6.13 [1.98–26.9], the second survey OR: 18.3 [1.07–312.9]), and recommendations from people close to parents (the first survey OR: 14.3 [4.99–52.0], the second survey OR: 28.7 [7.26–192.0]) were the factors associated with vaccine hesitancy that were above our cut‐off limit.

**TABLE 3:** Factors based on the 3Cs model associated with hesitation regarding COVID‐19 vaccination of children in Japan, 2022 (*n* = 134)

	First survey		Second survey		First survey	Second survey
	Acceptance group, *n* = 26 (%)	Hesitancy group, *n* = 108 (%)	Acceptance group, *n* = 15 (%)	Hesitancy group, *n* = 119 (%)	OR^a^ (95% CI)	OR^a^ (95% CI)
Confidence						
Vaccine is safe						
No/probably no	2 (7.7)	70 (64.8)	2 (13.3)	78 (65.5)	22.1 (6.12–142.3)	12.4 (3.22–81.5)
Yes/probably yes	24 (92.3)	38 (35.2)	13 (86.7)	41 (34.5)	Ref	Ref
Vaccine has an effect on COVID‐19						
No/probably no	1 (3.8)	39 (36.1)	1 (6.7)	51 (42.9)	14.1 (2.82–257.2)	10.5 (2.01–193.4)
Yes/probably yes	25 (96.2)	69 (63.9)	14 (93.3)	68 (57.1)	Ref	Ref
Vaccine is managed and supplied safely						
No/probably no	2 (7.7)	35 (32.4)	1 (6.7)	39 (32.8)	5.51 (1.52–35.5)	6.83 (1.30–125.9)
Yes/probably yes	24 (92.3)	73 (67.6)	14 (93.3)	80 (67.2)	Ref	Ref
Agree with government policy						
No/probably no	3 (11.5)	48 (44.4)	0 (0)	44 (37.0)	6.13 (1.98–26.9)	18.3 (1.07–312.9)
Yes/probably yes	23 (88.5)	60 (55.6)	15 (100)	75 (63.0)	Ref	Ref
Recommendations from people close to parents						
No/probably no	4 (15.4)	78 (72.2)	2 (13.3)	97 (81.5)	14.3 (4.99–52.0)	28.7 (7.26–192.0)
Yes/probably yes	22 (84.6)	30 (27.8)	13 (86.7)	22 (18.5)	Ref	Ref
Trust information from TV						
No/probably no	7 (26.9)	50 (46.3)	1 (6.7)	52 (43.7)	2.34 (0.94–6.41)	10.87 (2.08–200.07)
Yes/probably yes	19 (73.1)	58 (53.7)	14 (93.3)	67 (56.3)	Ref	Ref
Trust information from SNS						
No/probably no	20 (76.9)	81 (75.0)	7 (46.7)	79 (66.4)	0.90 (0.30–2.37)	2.26 (0.76–6.87)
Yes/probably yes	6 (23.1)	27 (25.0)	8 (53.3)	40 (33.6)	Ref	Ref
Trust information from people close to parents						
No/probably no	9 (34.6)	35 (32.4)	1 (6.7)	36 (30.3)	0.91 (0.37–2.31)	6.07 (1.15–112.1)
Yes/probably yes	17 (65.4)	73 (67.6)	14 (93.3)	83 (69.7)	Ref	Ref
Trust information from HCPs						
No/probably no	0 (0)	7 (6.5)	0 (0)	10 (8.4)	3.92 (0.22–70.8)	2.97 (0.17–53.3)
Yes/probably yes	26 (100)	101 (93.5)	15 (100)	109 (91.6)	Ref	Ref
Complacency						
Prevention measures are adequate in group life (e.g. day‐care and after‐school center)						
No/probably no	4 (15.4)	8 (7.4)	1 (6.7)	8 (6.7)	0.44 (0.13–1.77)	1.01 (0.17–19.4)
Yes/probably yes	22 (84.6)	100 (92.6)	14 (93.3)	111 (93.3)	Ref	Ref
Worry about the likelihood of getting COVID‐19 in group life						
No/probably no	4 (15.4)	7 (6.5)	1 (6.7)	14 (11.8)	0.38 (0.11–1.56)	1.87 (0.33–35.1)
Yes/probably yes	22 (84.6)	101 (93.5)	14 (93.3)	105 (88.2)	Ref	Ref
Worry about the likelihood of getting COVID‐19 in non‐group life						
No/probably no	12 (46.2)	54 (50.0)	5 (33.3)	54 (45.4)	1.17 (0.49–2.79)	1.66 (0.55–5.61)
Yes/probably yes	14 (53.8)	54 (50.0)	10 (66.7)	65 (54.6)	Ref	Ref
Worry about my child will get severe symptoms						
No/Probably no	16 (61.5)	65 (60.2)	9 (60.0)	75 (63.0)	0.94 (0.38–2.25)	1.14 (0.36–3.37)
Yes/Probably yes	10 (38.5)	43 (39.8)	6 (40.0)	44 (37.0)	Ref	Ref
Convenience						
The time for vaccination is appropriate^b^						
No/probably no			0 (0)	19 (20.4)		4.98 (0.28–90.3)
Yes/probably yes			8 (8.6)	66 (71.0)		Ref
The place for vaccination is appropriate^b^						
No/probably no			0 (0)	15 (16.1)		3.74 (0.20–68.2)
Yes/probably yes			8 (8.6)	70 (75.3)		Ref

Abbreviations: %, percentage; CI, confidence interval; COVID‐19, coronavirus disease; HCPs, Health care professionals; HPV, Human papillomavirus; *n*, number; OR, Odds ratio; SNS, Social networking service.

^a^ Higher odds ratio values indicate more hesitation.

^b^ Asked only for parents who have a child over 5 years old after starting vaccination for them (*n* = 93).

### Qualitative findings

In total, 10 parents took part in interviews (Figure 1) and data saturation was reached. Participants were eight mothers and two fathers aged 31–47 years. Eight parents were in the hesitancy group in both surveys, whereas two parents changed from the hesitancy to the acceptance group. Interviews lasted approximately 25–30 min.

The thematic analysis revealed five themes regarding hesitation related to COVID‐19 vaccination for children, namely: (i) Vaccination is bad for children's health, (ii) low necessity, (iii) dissatisfaction with policies, (iv) no encouragement from others, and (v) uncertain information (Table 4). Many parents reported that vaccination was bad for children's health. Some parents felt dissatisfaction with government policies. Encouragement from others, whether or not to vaccinate, was important. An ongoing rapport with HCPs was also essential for parental decision‐making. Many factors relating to obtaining information were reported in interviews. Many parents gave the fact that information around childhood vaccination was insufficient and uncertain as a reason for remaining undetermined. However, if parents felt they received sufficient reliable information, such as clinical trial results, they planned to change their mind from hesitancy to accepting vaccination for their children.

**TABLE 4:** Qualitative findings about the hesitation of COVID‐19 vaccination for children

**Theme 1 Vaccination is bad for children's health**
Subtheme 1 Concern about long‐term unknown side effects
Subtheme 2 Concern about side effects experienced by parents
Subtheme 3 Concern about aggravation of children's allergies
**Theme 2 Low necessity**
Subtheme 1 Unreliable effectiveness
Subtheme 2 Low risk of severe symptoms
Subtheme 3 Low infection threat
**Theme 3 Dissatisfaction with policies**
Subtheme 1 Ambiguous explanation from government authorities
Subtheme 2 Lack of convenience
Subtheme 3 Voluntary and insufficient compensation
**Theme 4 No encouragement from others**
Subtheme 1 No encouragement from close personal contacts
Subtheme 2 No encouragement from trusted HCPs
**Theme 5 Uncertain information**
Subtheme 1 Insufficient information about childhood vaccination
Subtheme 2 Uncertainty of information about childhood vaccination
Subtheme 3 Incomprehensible information

Abbreviation: HCPs, Health care professionals.

### Integration of findings

Table 1 shows the integrated qualitative and quantitative results. The left column shows vaccine hesitancy factors from quantitative surveys. The middle column depicts qualitative quotes from interviews. The right column shows integrated meta‐inferences. In addition to quantitative findings about vaccine safety, vaccine effectiveness, government policy, and recommendations from people close to parents, we discovered more detailed and nuanced perceptions during interviews.

Parents were concerned about vaccine safety because of unknown long‐term side effects and the vaccine's short‐term effectiveness. They were also skeptical of government policies due to ambiguous explanations. When people close to parents, such as family, did not encourage vaccination, parents did not tend to vaccinate their children. The quantitative study results show that when parents believed in safe and appropriate management of vaccination and supply of vaccines, it mitigated parental vaccine hesitancy. However, this was not included in the integration because it was not mentioned by parents during the interviews.

## DISCUSSION

**FIGURE 2:**
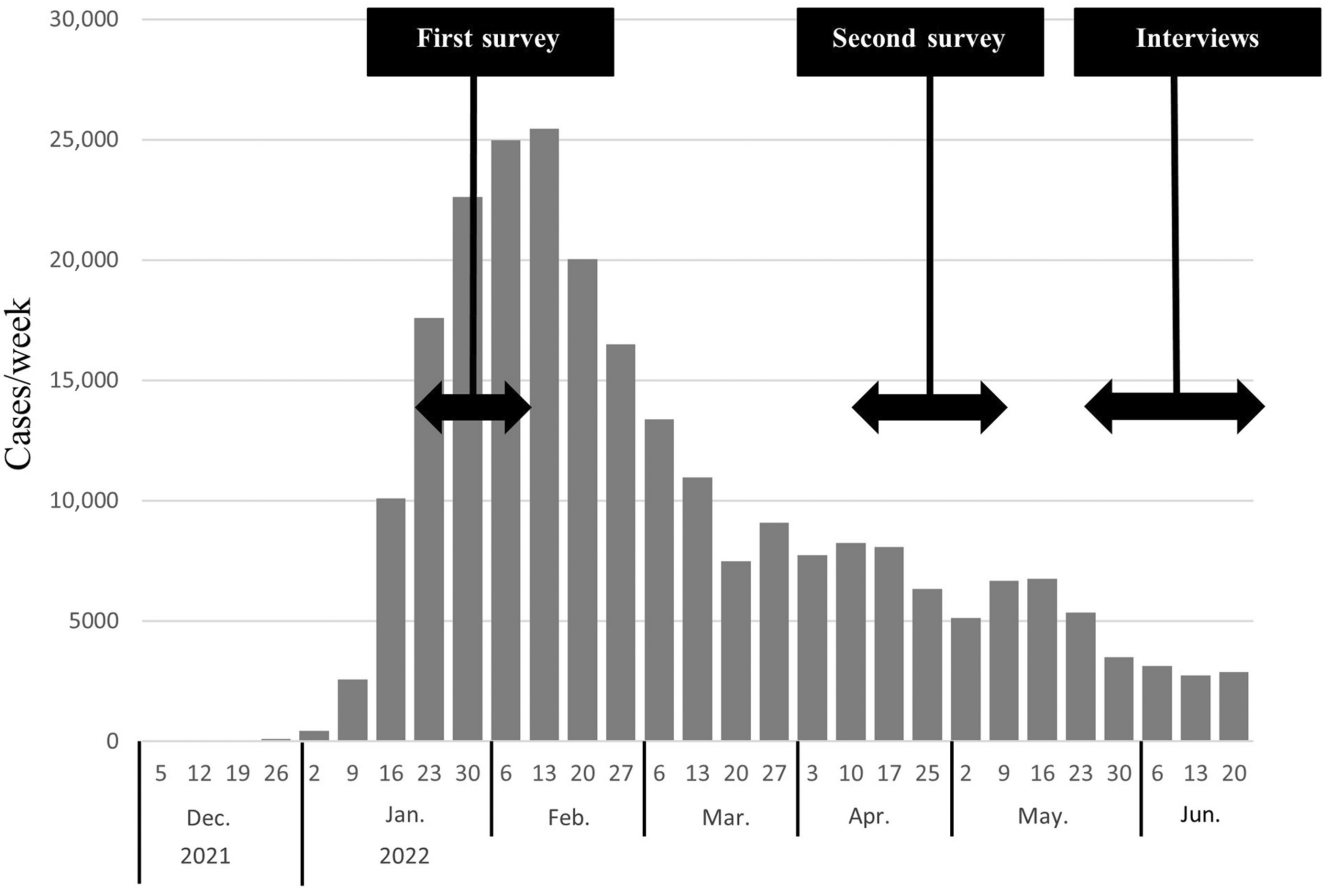
Number of new COVID‐19 cases in children aged under 10 in Osaka^31^. Note that only the number of new COVID‐19 cases in Osaka are shown as data was unavailble for Kyoto prefecture.

This study aimed to examine COVID‐19 vaccination acceptance and hesitancy among Japanese parents during a 3 month period around the peak of the pandemic (Figure 2),^31^ before and after the introduction of the vaccines for children. It also aimed to explore influencing factors. The mixed methods design of this study revealed several novel factors driving hesitancy including the short timeframe of vaccine rollout, perceived one‐sided information about risks and benefits, lack of information about vaccination safety from government, lack of clear justification for vaccination, and ambiguous policies around COVID‐19 vaccination and COVID‐19 countermeasures. Trust was a common theme determining decision‐making, particularly in the case of HCPs where parents indicated that clear and detailed explanations from familiar HCPs, and case‐by‐case consideration of factors such as allergies, influenced their choices. Finally, the longitudinal design of this study demonstrates changes in parental vaccine acceptance and hesitancy, with hesitancy increasing throughout the study despite ongoing COVID‐19 infections. The COVID‐19 vaccination acceptance rates were 19.4% (26/134) (95% CI: 12.7–26.1) in the first survey and 11.2% (15/134) (95% CI: 5.9–16.5) in the second survey. The change to hesitancy rate was 61.5% (16/26) (95% CI: 42.8–80.2), and the change to acceptance rate was 4.6% (5/108) (95% CI: 0.7–8.6).

Acceptance rates in this study are lower than in studies of parents' acceptance rates in other countries, which have a wide range of approximately 20% to 90%,^14^, ^15^, ^16^ as well as previous studies conducted among Japanese parents, which reported 42.9%^2^ and 64.7%.^3^ These acceptance rates might be affected by factors such as the pandemic situation, vaccine supply status,^14^ and the representativeness of the sample.

Despite the small sample size limiting our ability to identify factors influencing changes in acceptance and hesitancy, we were able to discover factors contributing to vaccine hesitancy through both quantitative and qualitative analysis. Our study aligned with previous studies in indicating that vaccine safety was the most important factor.^3^, ^14^, ^16^, ^17^, ^21^, ^26^ It also revealed that the relatively short time frame of vaccine development and distribution, in comparison with that of previous vaccines, was a trigger for parents' safety concerns. Many parents reported a lack of information about safety in the interviews. In a recent study, children's fever and chills incidence was lower than adults.^32^ However, this information may not have been readily available for parents during their decision‐making.^33^ In our study, therefore, parents likely assessed risk of children's side effects based on vaccination side effects that they had experienced themselves and that had been experienced by people close to them. In agreement with previous studies,^33^, ^34^ results from this study also indicated that information that only included the benefits of vaccination increased parental hesitancy. Thus, more balanced information, including both risks and benefits, about children may assist parental decision‐making.

Dissatisfaction with policies was another important factor, also seen in previous studies.^35^, ^36^, ^37^ Japan has some of the most negative attitudes toward governmental actions against COVID‐19, largely due to perceived ambiguous policies.^38^ Our results indicated ambiguous government explanations, including compensation for vaccination, might be one factor contributing to vaccine hesitancy. Understating or exclusion of detailed explanations of vaccination risks by government authorities, in the interest of preventing public apprehension, can have the opposite effect—increasing distrust.^39^ Past HPV vaccination measures also influenced COVID‐19 vaccine hesitancy. The Japanese government announced the withdrawal of proactive recommendations for HPV vaccination in 2013 because of safety concerns; however, they resumed recommendations in November 2021. We found that justifications for the government's recommendation policy were not clear to parents, leading to a general distrust of government measures. Consequently, even with limited evidence, provision of timely and accurate information in pandemics is important.^38^, ^39^ Additionally, in some cases, messages from government provoked “anti‐establishment” beliefs,^40^ suggesting advice from non‐government sources may be beneficial.

We found that recommendations from people close to parents, related to a general distrust and dissatisfaction with government policy, was an important factor in vaccination decisions. When people close to parents had negative attitudes toward vaccination, parents also changed their minds. Previous studies also indicated that parents required vaccination information from sources other than government/public organizations and public news media.^3^, ^33^ However, there are few previous studies conducted based on community‐wide sentiment,^41^ so further research on communities and like‐minded groups might be worthwhile.

Information from HCPs particularly affects parents.^12^, ^16^, ^33^ Trust in information from HCPs was not a vaccine hesitancy factor in quantitative surveys but our interviews revealed that trusted HCPs were important. In particular, our study showed parents tended to hesitate regarding vaccination if their children had allergies. Parents likely required information tailored to their children. However, previous studies mentioned physicians did not spend enough time discussing children's vaccine‐related issues with parents during appointments.^33^, ^42^ To help parents' decision‐making, they might require more discussion with familiar HCPs rather than just HCP recommendations through the media. This would also help individual parents who have specific concerns, such as children's allergies.^33^, ^43^

Finally, many factors about information were not included in the questionnaire but were reported in interviews. Some parents mentioned they wanted their child to be vaccinated when they received enough reliable information, such as clinical trial results. However, parents had difficulty in understanding vaccine‐related information and judging its trustworthiness.^33^ A previous study mentioned that health experts should aim to write easy‐to‐understand messages.^44^ Consequently, further research might be needed on how health authorities might provide information in a more comprehensible manner.

There are limitations to this study. First, the sample was restricted to certain regions in Japan. The majority of the study was located in Osaka city, which had one of the highest rates of infection within Japan during the study timeframe. Coronavirus disease 2019 countermeasures and official communications were largely determined by local governments and they varied by region. It is possible that these factors affected parents' decision‐making. This study was limited to parents with children attending center‐based care; they were more likely to be employed and have higher annual incomes in comparison with the general population.^45^ The study therefore has limited generalizability to the wider population of Japan. Second, we had to stop recruitment before our planned sample size was reached because the circumstances surrounding vaccination were changing rapidly and we aimed to maintain homogeneity in the collected information. However, the sample size achieved (134 instead of the planned 185) still allowed us to achieve reasonable precision in the prevalence estimates within ±10%, as the vaccination refusal rate was estimated to be 32.8% (95% CI: 24.9 to 40.8) in the first survey or 47.0% (95% CI: 38.6 to 55.5) in the second survey. Third, we did not restrict surveys to one person per family. Thus, perspectives were likely to be similar if there were multiple responses within the same family.

This study is subject to selection bias as the survey data were collected online and by volunteer participants. In this case, this study might include only parents who could access the Internet and were interested in this study's purpose. However, we could identify parental vaccine hesitancy factors in more detail by using a mixed‐methods approach and it can be useful to help parental decision‐making.

This study was also subject to reporting bias because parents self‐reported. Although they might give socially desirable responses, this study was conducted anonymously to minimize this bias. Finally, we did not ask parents about their children's COVID‐19 infection history. Although both questionnaire surveys were conducted during the sixth wave of the pandemic by the Omicron variant, we started the first survey before the peak in new infections, whereas the second survey was conducted after it. At the beginning of the epidemic, the proportion of pre‐teenage children infected was low; however, after January 2022, it increased to about 30%.^46^ Thus, during the timeframe of our study, it is likely that many participating children contracted COVID‐19. This may have influenced their decision‐making.^47^ However, we excluded this question to avoid causing parental fear of discrimination and prejudice.

## CONCLUSION

Overall, this study reveals the complexities of parental decision‐making around childhood vaccination in Japan. While our study showed that complacency and convenience were not major factors in parents' decision‐making, most parents hesitated for COVID‐19 vaccination for their children because of concerns about vaccine safety, vaccine effectiveness, government policy, and recommendations from people close to parents. These result aligned with confidence of the 3Cs model. Parents required readily available and more balanced information, including both risks and benefits, for their decision‐making, and further investigation is required to find ways to provide information that is easier for parents to understand. To support parental decision‐making, we suggest community‐wide support and discussions with familiar HCPs are of critical importance. Further research is needed to extend these findings to the wider Japanese population. However, the current study shows the importance of qualitative approaches, which can reveal novel hesitancy factors and provide a more nuanced understanding of how they drive parental decision‐making.

## AUTHOR CONTRIBUTIONS

Madoka Lelliott, Masatsugu Sakata, Ayako Kohno, and Toshi A Furukawa designed the study. Madoka Lelliott and Ayuko Matsumoto managed recruitment and data collection. Madoka Lelliott, Masatsugu Sakata and Rie Toyomoto analyzed the data and prepared the first draft of the manuscript. All authors contributed to interpretations of the findings, the study design and approved the final version of the manuscript.

## FUNDING INFORMATION

This study was funded by Kyoto University Medical Student and Researcher Support‐Fund (KMS‐FUND).

## CONFLICT OF INTEREST STATEMENT

Toshi A Furukawa reports personal fees from Boehringer‐Ingelheim, DT Axis, Kyoto University Original, Shionogi, and SONY, and a research grant from Shionogi. Toshi A Furukawa has patents 2020‐548587 and 2022‐082495 pending, and intellectual properties for the Kokoro application licensed to Mitsubishi‐Tanabe. All the other authors declare no competing interests.

## ETHICS STATEMENT

Ethical approval was obtained from Kyoto University Graduate School and Faculty of Medicine ethics committee (approval number: R3290).

## Supporting information


Appendix S1.

Appendix S2.
