# Supplementary material for: Perspectives on childhood coronavirus disease vaccination in Japan and influencing factors
Source: Pediatr Int. 2024 Sep 30;66(1):e15819. doi: 10.1111/ped.15819 (PMC11580370; doi:10.1111/ped.15819)
Supplement: Supplementary file 1 — Appendix S1. [file PED-66-e15819-s002.docx]

Supplementary File 1: Questionnaire (English version)

**Ⅰ.　About COVID-19 and the vaccine**

|  | Yes Probably Probably No  Yes No |
| --- | --- |
| 1. Do you think the COVID-19 vaccine is safe for your children? | □　　　　　　□　　　　　　□　　　　　　□ |
| 2. Do you think the COVID-19 vaccine is effective for children? | □　　　　　　□　　　　　　□　　　　　　□ |
| 3. Do you think the COVID-19 vaccine is managed and supplied safely? | □　　　　　　□　　　　　　□　　　　　　□ |
| 4. Do you agree with government policy? | □　　　　　　□　　　　　　□　　　　　　□ |
| 5. Do you think prevention measures are adequate in group life (e.g. day-care and after-school center)? | □　　　　　　□　　　　　　□　　　　　　□ |
| 6．Do you worry about the likelihood of getting COVID-19 in group life (e.g. day-care and after-school center)? | □　　　　　　□　　　　　　□　　　　　　□ |
| 7．Do you worry about the likelihood of getting COVID-19 in non-group life? | □　　　　　　□　　　　　　□　　　　　　□ |
| 8．Do you worry your child will get heavy symptoms? | □　　　　　　□　　　　　　□　　　　　　□ |
| 9．Do you think the time for vaccination is appropriate? | □　　　　　　□　　　　　　□　　　　　　□ |
| 10．Do you think the place for vaccination is appropriate? | □　　　　　　□　　　　　　□　　　　　　□ |
| 11. Do you get any recommendations from people close to parent (e.g. family, relatives and friends)? | □　　　　　　□　　　　　　□　　　　　　□ |
| 12.　 Do you trust information from TV? | □　　　　　　□　　　　　　□　　　　　　□ |
| 13.Do you trust information from SNS (e.g. blogs, Twitter, Instagram, Facebook and YouTube)? | □　　　　　　□　　　　　　□　　　　　　□ |
| 14.　Do you trust information from your friends and family members? | □　　　　　　□　　　　　　□　　　　　　□ |
| 15．Do you trust information from HCPs (e.g. doctors and nurses)? | □　　　　　　□　　　　　　□　　　　　　□ |

| Do you want your child to get COVID-19 vaccination if it is available? |
| --- |
| □Yes　　　　□　Undecided　　　□　No  Please feel free to write the reasons: |

**Ⅱ.　About other vaccines**

|  | Yes Probably Probably No  Yes No |
| --- | --- |
| 1． In general, do you think it's a good idea for children to get a influenza vaccination every year? | □　　　　　　□　　　　　　□　　　　　　□ |
| 2． In general, do you think it's a good idea for children to get a mumps vaccination? | □　　　　　　□　　　　　　□　　　　　　□ |
| 3．In general, do you think it's a good idea for children to get a HPV vaccination? | □　　　　　　□　　　　　　□　　　　　　□ |

**Ⅲ. About your child**

| 1. How many siblings does he/she have? | □　One　　　　□　Two　　　　　　 □　More than three |
| --- | --- |
| 2. Is he/she your first/second/third child? | □　First □　Second □　More than third |
| 3. How old is he/she? | (　　　　　　　　　) month　　or　(　　　　　　　) years old |
| 4. What is his/her gender? | □　boy　　　　　 □ girl　　　　　 □　others |
| 5．Does he/she have a chronic disease? | □　Yes □　No |
| 6. Have he/she received all available vaccinations? | □　Yes □　No |
| 7. Did he/she received the influenza vaccination since October last year? | □　Yes □　No |

**Ⅳ.　About yourself**

| 1. What is your relationship with your child? | □　Mother　　　　　　　　　　□　Father  □　Others　（　　　　　　　　　　　　　　　　　　　　　　　　　　　　　） |
| --- | --- |
| 2. How old are you? | (　　　　　　　　) years old |
| 3．What is the highest level of education you have completed? | □ Graduate school □ University  □ Junior college  □　High school □　Junior high school  □　Others （　　　　　　　　　　　　　　　　　　　　　　　　　　　　　　） |
| 4. Are you working? | □　Yes　　　　　□　No  □　Others（　　　　　　　　　　　　　　　　　　　　　　　　　　　　　　） |
| 5． Is there anyone in your household who is a healthcare worker? | □　Yes □　No |
| 6. Is there anyone in your household who is an educator? | □　Yes □　No |
| 7．Did you get a COVID-19 vaccination? | □　Yes □　No |
| 8. Did you receive an influenza vaccination since October last year? | □　Yes □　No |
