# Supplementary material for: Perspectives on childhood coronavirus disease vaccination in Japan and influencing factors
Source: Pediatr Int. 2024 Sep 30;66(1):e15819. doi: 10.1111/ped.15819 (PMC11580370; doi:10.1111/ped.15819)
Supplement: Supplementary file 2 — Appendix S2. [file PED-66-e15819-s001.docx]

**Supplementary File 2:** Interview guide (English Ver.)

| Contents | Detail |
| --- | --- |
| ＜Introduction＞  How was the questionnaire survey? | ➢Was it easy to answer?  ➢What did you think about the surveys? |

| Contents | Detail |
| --- | --- |
| Please let me know what you think about COVID-19 vaccination. | ➢ Please let me know about the［factor］in detail  ➢What is the most important thing for considering vaccination for your child  ➢Please let me know in detail about your comments in the survey  ➢How did you get the information  ➢What kind of information did you get |
| 【Change】  Have you had any experience thinking about [factor]? What was your experience like? | ➢What kind of information did you get  ➢What kind of changes have occurred  ➢What do you think now  ➢What did you think before  ➢What do you need more of |
| 【No Change】 | ➢What kind of things/experiences do you need to decide whether to get vaccination or not for your child  ➢What kind of information do you need  ➢What kind of situation do you need  ➢What kind of change do you need  ➢How do you judge this  ➢Let me know more detail |
| Please let me know if you have anything else |  |
